# The mediating role of social support in the association between organizational climate and presenteeism among endoscopy nurses: a multicenter cross-sectional study

**DOI:** 10.3389/fpubh.2026.1848943

**Published:** 2026-06-10

**Authors:** Xue Zhou, Xingzi Chen, Kangrui Wang, Jiani Qu, Haoyu Zou

**Affiliations:** 1 The First Hospital Affiliated to Hunan Normal University (Hunan Provincial People's Hospital), Changsha, Hunan, China; 2 School of Nursing, Hunan Normal University, Changsha, Hunan, China; 3 Hunan Provincial People's Hospital (The First Hospital Affiliated to Hunan Normal University), Changsha, Hunan, China

**Keywords:** endoscopy nurses, job demands-resources model, organizational climate, presenteeism, social support

## Abstract

**Objective:**

This study aimed to investigate the mediating role of social support in the association between organizational climate and presenteeism among endoscopy nurses. This research provides an empirical and theoretical foundation for addressing presenteeism in clinical practice.

**Methods:**

This multicenter cross-sectional study enrolled 225 endoscopy nurses from 20 hospitals across Hunan Province, China, between April and May 2025. Participants completed a sociodemographic questionnaire, the Organizational Climate Questionnaire, the Social Support Rating Scale, and the Stanford Presenteeism Scale-6 (SPS-6). Hierarchical multiple regression analysis was conducted to identify factors associated with presenteeism, adjusting for self-reported physical health status and the provision of protective equipment. Additionally, a bias-corrected Bootstrapping approach was utilized to evaluate the hypothesized mediating effects.

**Results:**

Endoscopy nurses reported mean scores of 72.89 ± 17.66 for organizational climate, 39.97 ± 9.67 for social support, and 16.83 ± 5.81 for presenteeism. Correlation analyses demonstrated that organizational climate was positively associated with social support and negatively associated with presenteeism (*p* < 0.01). Hierarchical multiple regression analysis revealed that physical health status, provision of protective equipment, organizational climate, and social support were significantly associated with presenteeism (*p* < 0.05). Mediation analysis revealed that social support partially mediated the association between organizational climate and presenteeism. The standardized indirect effect was −0.105 (95% CI: −0.197, −0.026, *p* = 0.005), accounting for 28.85% of the total effect.

**Conclusions:**

The findings indicate that a positive organizational climate is inversely associated with presenteeism, and this association is partially mediated by social support among endoscopy nurses. Nursing administrators should cultivate a supportive organizational environment and strengthen social support networks, as these strategies may contribute to the better management of presenteeism behaviors in clinical settings.

## 1 Introduction

According to the latest global cancer statistics (GLOBOCAN 2020), the incidence of gastrointestinal malignancies ranks among the top five globally (1). As digestive endoscopy is widely recognized as the gold standard for the early screening and diagnosis of these malignancies, the clinical workload and procedural volume in endoscopy centers have increased significantly (2, 3).

Presenteeism is defined as the phenomenon where employees continue to work despite experiencing physical or mental unwellness (4). For nursing professionals, presenteeism is not only associated with depleted physiological and psychological resources, but also presents substantial risks to patient safety (5). A recent meta-analysis found that the global prevalence of presenteeism among nurses is approximately 49.2%, whereas studies in China have reported prevalence rates exceeding 70%. This phenomenon may be partly attributed to acute workforce shortages (6, 7).

To manage these occupational risks, job resources such as social support and organizational climate are considered crucial (8). Social support is a multidimensional construct encompassing tangible assistance, emotional validation, and the active utilization of social networks (9). Empirical studies have demonstrated that higher levels of social support are negatively correlated with presenteeism, as the effective utilization of support networks empowers nurses to seek help and recover rather than working while ill (10). When nurses possess robust social support, the integration of material aid and emotional support may facilitate the replenishment of their psychological capital, which is linked to lower physiological costs associated with stress adaptation (11, 12). Furthermore, organizational climate is defined as the shared collective perceptions among employees concerning the policies, practices, and established procedures within their institution (13). It operates as a fundamental structural job resource that not only theoretically buffers against the effects of job demands, but also functions as a proximal mechanism facilitating the efficacy of other job resources (14).

Despite the critical implications of these factors, research exploring the underlying interaction mechanisms among organizational climate, social support, and presenteeism remains limited, and studies specifically targeting the unique, high-throughput environment of endoscopy nurses are even more scarce. Distinct from general ward environments, endoscopy practice is characterized by a highly intensive and procedurally driven workflow (15). Nurses navigate specific occupational stressors, including prolonged standing in radiation protective gear, chemical exposure, and biological hazards (16, 17). Furthermore, routine manual abdominal compressions impose unique ergonomic burdens (18). Operationally, the reliance on tightly coupled clinical teams contributes to substantial interprofessional dependency, which can magnify the disruptive impact of unscheduled absences (19). In such settings, taking legitimate medical leave may place additional operational pressure on remaining colleagues. These structural and operational pressures may inadvertently contribute to presenteeism. While previous research among general nursing populations frequently examines organizational climate and social support as independent correlates of occupational health, the potential sequential relationship between these resources has received less attention (20). To provide a more comprehensive understanding, this study extends current literature by exploring its mediating mechanism.

**Figure 1:**
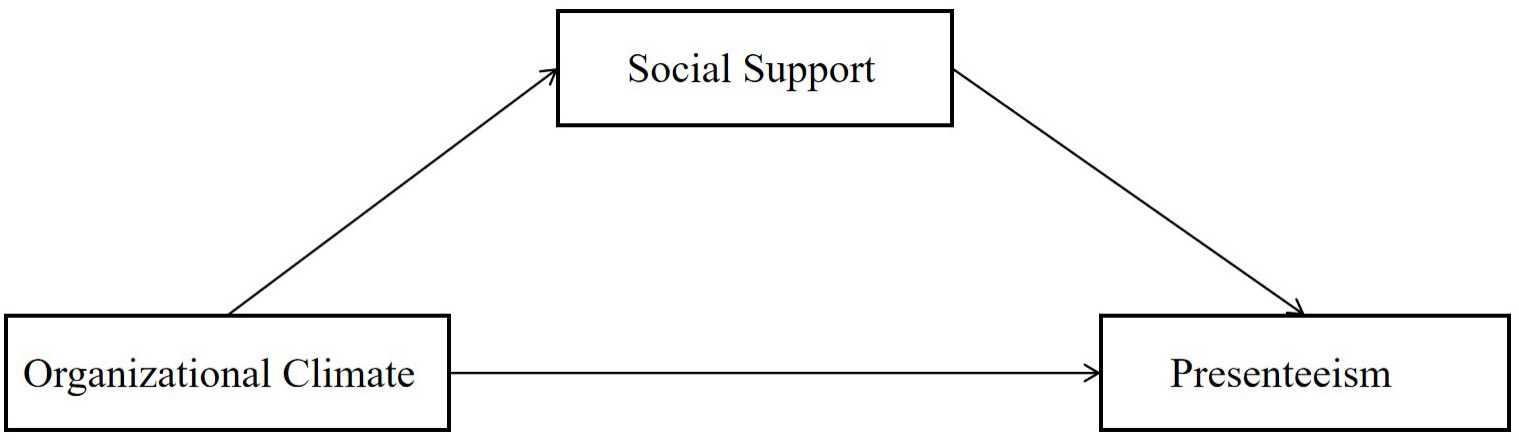
Theoretical model of the mediating role of social support between organizational climate and presenteeism.

Guided by the Job Demands-Resources model (8), we propose that organizational climate, functioning as a macro-level structural resource, may facilitate the accumulation of social support, a micro-level interpersonal resource. Examining this sequential pathway is particularly relevant in the tightly coupled, team-driven environment of endoscopy, where effective peer support appears to be closely linked to a healthy departmental climate (21). Consequently, this study designates organizational climate as the independent variable, social support as the mediating variable, and presenteeism as the dependent variable, proposing the following hypotheses: Hypothesis 1: organizational climate is negatively associated with presenteeism among endoscopy nurses; Hypothesis 2: social support is negatively associated with presenteeism; and Hypothesis 3: social support mediates the association between organizational climate and presenteeism (Figure 1). By elucidating these mechanisms, this study seeks to provide nursing managers with targeted evidence to optimize departmental climates and construct robust social support systems, thereby supporting the management of presenteeism and the safeguarding of patient care quality.

## 2 Materials and methods

### 2.1 Study design and participants

A cross-sectional descriptive design was employed. Between April and May 2025, participants were recruited from 20 hospitals across Hunan Province, China. To ensure a diverse representation of the nursing workforce, a purposive-convenience sampling approach was utilized to select the institutions. The 20 hospitals were chosen based on geographical accessibility, established collaborative networks, and the need to include various healthcare tiers, encompassing 10 Tertiary Level A hospitals, 4 Tertiary Level B hospitals, and 6 Secondary level and below hospitals. Among the 225 valid responses, 177 participants were from tertiary hospitals, and 48 were from secondary hospitals or below, ensuring a representative distribution of the endoscopy nursing workforce across different healthcare tiers.

According to Kendall's guidelines for sample size estimation, the required number of participants should be five to ten times the number of questionnaire items (22). Based on the 33 items across the study instruments, the baseline sample size was estimated to be between 165 and 330 participants. To account for a potential 20% invalid response rate, the target sample size was adjusted to a range of 207 to 413. During the data screening of the 240 initial responses, 15 submissions were excluded due to completion times of less than 3 min or the presence of systematic response patterns. Ultimately, 225 valid questionnaires were retained for statistical analysis, yielding an effective response rate of 93.75% and fulfilling the predetermined sample size requirements.

Inclusion criteria: (1) participants held a valid registered nurse license; (2) they had at least 1 year of clinical experience in digestive endoscopy nursing; (3) they provided informed consent and voluntarily agreed to participate in the study.

Exclusion criteria: (1) participants were rotating nurses or were currently enrolled in short-term training programs; (2) they had taken cumulative leave of absence, such as maternity or personal leave, exceeding 3 months within the preceding year; (3) they had a diagnosed psychiatric disorder.

Ethical approval for this study was granted by the Ethics Committee of the Medical College at Hunan Normal University (2025.490).

### 2.2 Research tools

#### 2.2.1 General demographic questionnaire

An investigator-developed sociodemographic questionnaire, refined through consultations with clinical experts in digestive endoscopy, was used to collect participant data. This instrument assessed demographic and occupational characteristics, including gender, age, marital status, educational attainment, monthly income, hospital classification, professional title, self-reported physical health status, and the adequacy of protective equipment provision.

#### 2.2.2 Presenteeism scale

Presenteeism was evaluated using the Chinese version of the 6-item Stanford Presenteeism Scale (SPS-6). This instrument was originally developed by Koopman (22, 23) and culturally adapted into Chinese by Zhao (24). The SPS-6 assesses health-related productivity loss experienced by employees who continue to work despite illness. Items are rated on a 5-point Likert scale ranging from 1 (strongly disagree) to 5 (strongly agree). With items 5 and 6 being reverse-scored, total scores range from 6 to 30, where higher scores indicate greater levels of presenteeism. In the present study, the scale demonstrated good internal consistency, yielding a Cronbach's α coefficient of 0.834.

#### 2.2.3 Social support rating scale

Social support was assessed using the Social Support Rating Scale (SSRS), developed by Xiao (25). This 10-item instrument encompasses three dimensions: objective support (three items), subjective support (four items), and utilization of support (three items). The instrument employs varying scoring mechanisms across its items: items 1–4 and 8–10 are rated on a standard 4-point Likert scale; item 5 comprises four sub-items (A–D), each scored from 1 (no support) to 4 (full support), with their sum constituting the item's final score. For items 6 and 7, scores correspond directly to the number of support sources reported, whereby a score of 0 is assigned when no sources are selected, and the score increases incrementally with each additional source. The total score is calculated by summing all 10 items, where a higher total score indicates a greater level of perceived social support. In the present study, the scale demonstrated good internal consistency, yielding a Cronbach's α coefficient of 0.867.

#### 2.2.4 Organizational climate questionnaire

Organizational climate was evaluated using the Organizational Climate Questionnaire, developed by Chen (26). This 17-item instrument encompasses four distinct dimensions: hospital management (four items), departmental leadership and communication (five items), work collaboration and prospects (four items), and doctor-patient mutual understanding and communication (four items). Responses are rated on a 6-point Likert scale ranging from 1 (completely disagree) to 6 (completely agree). Total scores range from 17 to 102, with higher scores reflecting a more positive organizational climate. In the present study, the instrument demonstrated good internal consistency, yielding a Cronbach's α coefficient of 0.874.

### 2.3 Data collection

Data collection was conducted in the endoscopy centers of 20 general hospitals of various tiers in Hunan Province, China. Prior to data collection, formal authorization was obtained from hospital administrators and department heads. Head nurses at each participating center were trained on the study's objectives, protocols, and standardized guidelines for questionnaire administration. These head nurses subsequently identified eligible participants based on the predefined inclusion and exclusion criteria, distributing survey access via Quick Response codes. The survey was administered electronically via www.wjx.cn, an online survey platform in China. To minimize missing data and duplicate responses, all items were set as mandatory fields, and system settings restricted submissions to a single entry per IP address or WeChat account. Following data exportation, two researchers independently cleaned and cross-checked the dataset. Responses completed in under 3 min, or those exhibiting systematic or illogical answering patterns, were excluded to ensure data quality.

### 2.4 Statistical analysis

Statistical analyses were performed using SPSS 27.0 and AMOS version 26.0. The Kolmogorov-Smirnov test was utilized to assess the normality of the data. Normally distributed data were expressed as means and standard deviations (SD), and group comparisons were conducted using independent *t*-tests or one-way analysis of variance. For variables deviating from a normal distribution, medians and interquartile ranges were reported, and non-parametric tests were applied. Pearson correlation analysis was employed to examine the bivariate associations among organizational climate, social support, and presenteeism. To identify factors associated with presenteeism, hierarchical multiple regression analysis was conducted, adjusting for sociodemographic variables that demonstrated statistical significance in the univariate analyses. Harman's single-factor test was performed to evaluate potential common method bias. This study used AMOS to test whether social support mediates the association between organizational climate and presenteeism, and employed the Bootstrap method to analyze the mediating effect. A two-sided *p*-value < 0.05 was considered statistically significant.

## 3 Results

### 3.1 Demographic characteristics

As detailed in Table 1, the sample consisted of 225 endoscopy nurses. Participants were predominantly female (93.8%) and married (76.9%), with the majority aged between 31 and 50 years (68.9%). More than three-quarters of the participants (79.1%) held a bachelor's degree or higher. Professionally, most nurses were employed in Tertiary Level A hospitals (67.1%) and held the title of nurse-in-charge (52.4%). The cohort was relatively evenly distributed in terms of employment status, comprising permanent staff (46.7%) and contract-based employees (53.3%). Nearly half of the participants (46.7%) had less than 5 years of clinical experience in digestive endoscopy, and 63.1% of the nurses worked between 40 and 50 h per week. Regarding occupational health and safety variables, 64.0% of the respondents self-reported their physical health status as good, and 55.1% reported having access to fully equipped protective gear.

**Table 1:** Demographic and occupational characteristics of endoscopy nurses (*N* = 225).

Variable	Categories	Number of cases (*n*)	Percentage (%)
Gender	Male	14	6.2
	Female	211	93.8
Age (years)	≤ 30	53	23.5
	31–40	86	38.2
	41–50	69	30.7
	≥51	17	7.6
Marital status	Married	173	76.9
	Single	52	23.1
Number of children	0	46	20.4
	1	88	39.1
	≥2	91	40.5
Education level	Secondary vocational	5	2.2
	Junior college	42	18.7
	Bachelor's degree	169	75.1
	Master's and above	9	4.0
Hospital level	Tertiary level A	151	67.1
	Tertiary level B	26	11.6
	Secondary or below	48	21.3
Years of digestive endoscopy experience	< 5 years	105	46.7
	5–15 years	101	44.9
	16–25 years	15	6.7
	26–35 years	4	1.7
Professional title	Nurse	31	13.8
	Senior nurse	36	16.0
	Nurse-in-charge	118	52.4
	Associate chief nurse and above	40	17.8
Employment status	Permanent staff	105	46.7
	Contract-based	120	53.3
Health status	Good	144	64.0
	Fair	71	31.6
	Poor	10	4.4
Weekly work hours	< 40	50	22.2
	40–50	142	63.1
	>50	33	14.7
Protective equip.	Fully equipped	124	55.1
	Partially equipped	93	41.3
	Not equipped	8	3.6

CNY, Chinese Yuan.

### 3.2 Scores for organizational climate, social support, and presenteeism

As shown in Table 2, the endoscopy nurses reported a mean presenteeism score of 16.83 ± 5.81. Given the possible scale range of 6 to 30, this result indicates a moderate level of presenteeism among the cohort. The mean total score for organizational climate was 72.89 ± 17.66. Within this construct, departmental leadership and communication obtained the highest score (21.42 ± 5.82), whereas hospital management scored the lowest (16.19 ± 4.46). Additionally, participants reported a mean total score of 39.97 ± 9.67 for social support. Among its three dimensions, subjective support yielded the highest mean score (23.12 ± 6.11), followed by objective support (9.34 ± 3.18) and support utilization (7.51 ± 1.99).

**Table 2:** Scores for social support, organizational climate, and presenteeism among endoscopy nurses (*N* = 225).

Variable	Number of items	Total score (*M ±SD*)	Item (*M ±SD*)
**Presenteeism**	6	16.83 ± 5.81	2.81 ± 0.97
**Social support**	10	39.97 ± 9.67	3.99 ± 0.97
Subjective support	4	23.12 ± 6.11	5.78 ± 1.53
Objective support	3	9.34 ± 3.18	3.11 ± 1.06
Support utilization	3	7.51 ± 1.99	2.50 ± 0.67
**Organizational climate**	17	72.90 ± 17.66	4.29 ± 1.04
Department leadership & comm	5	21.42 ± 5.82	4.28 ± 1.16
Doctor-patient understanding & comm.	4	18.33 ± 4.00	4.58 ± 1.00
Work collaboration & prospects	4	16.96 ± 4.89	4.24 ± 1.22
Hospital management	4	16.19 ± 4.46	4.05 ± 1.12

*M*, mean; *SD*, standard deviation.

### 3.3 Differences in presenteeism across demographic and occupational characteristics

As presented in Table 3, univariate analyses revealed significant variations in presenteeism scores across specific demographic, occupational, and health-related categories. Presenteeism scores were significantly higher among nurses who were single (*p* = 0.001) and those without children (*p* = 0.009). Regarding institutional and occupational parameters, significantly higher presenteeism was reported by nurses employed in hospitals of Secondary level and below, individuals reporting fair or poor physical health, and those lacking fully equipped protective gear (all *p* < 0.001). Conversely, no statistically significant differences in presenteeism scores were observed across other examined variables, including gender, age, educational level, years of clinical experience in digestive endoscopy, professional title, employment status, and weekly working hours (all *p* > 0.05).

**Table 3:** Differences in presenteeism across demographic and occupational characteristics (*N* = 225).

Variable	Categories	Number of cases (*n*)	Percentage (%)	*M ±SD*	*t/F*	*p*
Gender	Male	14	6.2	18.50 ± 8.26	1.112	0.268
	Female	211	93.8	16.72 ± 5.62		
Age (years)	≤ 30	53	23.5	18.47 ± 7.03	2.212	0.088
	31–40	86	38.2	16.26 ± 5.55		
	41–50	69	30.7	16.09 ± 4.91		
	≥51	17	7.6	17.65 ± 5.71		
Marital status	Married	173	76.9	16.15 ± 5.37	3.273	0.001
	Single	52	23.1	19.09 ± 6.67		
Number of children	0	46	20.4	19.13 ± 6.98	4.798	0.009
	1	88	39.1	16.45 ± 5.11		
	≥2	91	40.5	16.03 ± 5.56		
Education level	Secondary vocational	5	2.2	17.60 ± 9.34	0.980	0.403
	Junior college	42	18.7	17.31 ± 6.02		
	Bachelor's degree	169	75.1	16.54 ± 5.56		
	Master's and above	9	4.0	19.67 ± 7.39		
Hospital level	Tertiary level A	151	67.1	15.74 ± 5.45	9.083	< 0.001
	Tertiary level B	26	11.6	18.11 ± 6.63		
	Secondary or below	48	21.4	19.54 ± 5.53		
Years of digestive endoscopy experience	< 5 years	105	46.7	16.79 ± 6.13	1.054	0.370
	5–15 years	101	44.9	17.25 ± 5.55		
	16–25 years	15	6.7	14.40 ± 5.50		
	26–35 years	4	1.7	16.50 ± 4.36		
Professional title	Nurse	31	13.8	18.23 ± 6.21	0.905	0.439
	Senior nurse	36	16	16.11 ± 5.63		
	Nurse-in-charge	118	52.4	16.57 ± 5.86		
	Associate chief nurse and above	40	17.8	17.16 ± 5.51		
Employment status	Permanent staff	105	46.7	16.18 ± 5.57	1.601	0.111
	Contract-based	120	53.3	17.41 ± 5.91		
Health status	Good	144	64	15.09 ± 5.52	21.243	< 0.001
	Fair	71	31.6	19.84 ± 4.74		
	Poor	10	4.4	20.50 ± 6.81		
Weekly work hours	< 40	50	22.2	15.96 ± 6.28	2.203	0.113
	40–50	142	63.1	16.72 ± 5.47		
	>50	33	14.7	18.64 ± 6.29		
Protective equip.	Fully equipped	124	55.1	14.95 ± 5.34	19.513	< 0.001
	Partially equipped	93	41.3	18.77 ± 5.13		
	Not equipped	8	3.6	23.38 ± 8.41		

### 3.4 Correlation analysis of organizational climate, social support, and presenteeism

As shown in Table 4, the Pearson correlation analysis revealed that both organizational climate (*r* = −0.398, *p* < 0.01) and social support (*r* = −0.383, *p* < 0.01) were significantly and negatively correlated with presenteeism. Additionally, a significant positive correlation was observed between organizational climate and social support (*r* = 0.583, *p* < 0.01).

**Table 4:** Correlation analysis of organizational climate, social support, and presenteeism among endoscopy nurses (*N* = 225).

Variables	Organizational climate	Social support	Presenteeism
Organizational climate	1		
Social support	0.583^**^	1	
Presenteeism	−0.398^**^	−0.383^**^	1

^**^p < 0.01.

### 3.5 Common method bias test

Potential common method bias was evaluated using Harman's single-factor test. An unrotated exploratory factor analysis incorporating all measurement items extracted nine distinct factors with eigenvalues exceeding 1. The primary factor explained only 35.81% of the total variance, remaining well below the conservative 40% threshold. These findings indicate that the results of this study are not substantially confounded by common method variance.

### 3.6 Testing assumptions of multiple regression

Prior to the hierarchical regression analysis, multicollinearity diagnostics were conducted with presenteeism as the dependent variable. The assessment revealed that the variance inflation factors (VIFs) for the dummy-coded categories of marital status, number of children, and hospital grade exceeded the prespecified threshold of 5 (Table 5), indicating significant multicollinearity. The high collinearity observed is likely attributable to the natural demographic overlap between marital status and the number of children, as well as the potential confounding between hospital grade and the allocation of occupational resources (27). Although theoretically relevant to presenteeism, their excessive VIFs suggest severe empirical redundancy. Methodologically, retaining highly collinear control variables may artificially inflate standard errors, which could compromise the accurate estimation of the study's core theoretical independent variables (28). Given their role as sociodemographic covariates rather than primary independent variables, these three variables were omitted from subsequent regression stages to preserve estimation accuracy and model validity. For all retained independent variables, the VIFs remained strictly within acceptable limits (maximum VIF = 2.17), confirming the absence of further multicollinearity concerns among the final independent variables. In addition to multicollinearity, other fundamental assumptions of regression analysis were thoroughly examined. The Durbin-Watson statistic was 1.975, indicating the independence of residual errors. Additionally, visual inspection of the Normal P-P plot of regression standardized residuals demonstrated that the data were normally distributed. The scatterplot of standardized residuals against predicted values confirmed that the assumptions of linearity and homoscedasticity were adequately met, with no obvious patterns or funneling observed.

**Table 5:** Multicollinearity diagnostics.

Variables	Categories	Tolerance	VIF
Marital status	Single	0.137	7.299
Number of children	1	0.186	5.382
	≥2	0.189	5.298
Hospital level	Tertiary Level B	0.168	5.952
	Secondary or below	0.151	6.623
Physical health status	Fair	0.795	1.257
	Poor	0.814	1.229
Protective equipment provision	Partially equipped	0.764	1.308
	Not equipped	0.762	1.312
Social support		0.461	2.169
Organizational climate		0.538	1.859

### 3.7 Hierarchical multiple regression analysis of presenteeism

As presented in Table 6, Model 1 incorporated demographic and occupational control variables, revealing that poorer physical health status and inadequate provision of protective equipment were significantly associated with higher presenteeism scores, accounting for 24.0% of the variance (*R*^2^ = 0.240, *p* < 0.001). The subsequent entry of organizational climate in Model 2 significantly increased the explanatory power by 3.0% (Δ*R*^2^ = 0.030, *p* = 0.003). Organizational climate exhibited a significant negative association with presenteeism (*B* = −0.067). In the fully adjusted final model (Model 3), the inclusion of social support contributed an additional 1.4% to the explained variance (Δ*R*^2^ = 0.014). Social support demonstrated a significant negative association with presenteeism (*B* = −0.085, *p* = 0.036), while organizational climate maintained its significant negative association (*B* = −0.045, *p* = 0.021). Overall, the comprehensive model explained 28.4% of the total variance in presenteeism (*R*^2^ = 0.284, Adjusted *R*^2^ = 0.264, *F* = 14.478, *p* < 0.001). Within this final model, poorer physical health, restricted access to protective equipment, lower organizational climate, and lower social support persisted as significant correlates of higher presenteeism.

**Table 6:** Hierarchical multiple regression analysis of factors associated with of presenteeism among endoscopy nurses (*N* = 225).

Variables	Intra-class independent variables	Model 1			Model 2			Model 3		
		*B*	95% CI	*p*	*B*	95% CI	*p*	*B*	95% CI	*p*
(Constant)		14.033	13.07, 15.00	< 0.001	19.512	15.78, 23.24	< 0.001	21.445	17.23, 25.66	< 0.001
Control variables										
Physical health	Fair	3.821	2.31, 5.33	< 0.001	3.245	1.72, 4.77	< 0.001	2.954	1.40, 4.50	< 0.001
	Poor	3.633	0.17, 7.09	0.041	2.856	−0.58, 6.29	0.105	2.273	−1.19, 5.74	0.200
Protective equipment	Partially equipped	2.898	1.48, 4.32	< 0.001	2.059	0.56, 3.56	0.008	2.071	0.58, 3.56	0.007
	Not equipped	6.546	2.68, 10.41	0.001	4.958	1.02, 8.90	0.014	4.234	0.25, 8.22	0.039
Organizational climate		—	—	—	−0.067	−0.110, −0.024	0.003	−0.045	−0.083, −0.007	0.021
Social support		—	—	—	—	—	—	−0.085	−0.164, −0.006	0.036
Model statistics										
	*F*	17.377		< 0.001	16.202		< 0.001	14.478		< 0.001
	*R*	0.49			0.519			0.533		
	*R^2^*	0.24			0.27			0.284		
	Adjusted *R^2^*	0.226			0.253			0.264		
	Δ *R^2^*	0.24			0.03			0.014		

CI, confidence interval. The dependent variable is presenteeism.

### 3.8 Mediating role of social support between organizational climate and presenteeism

**Figure 2:**
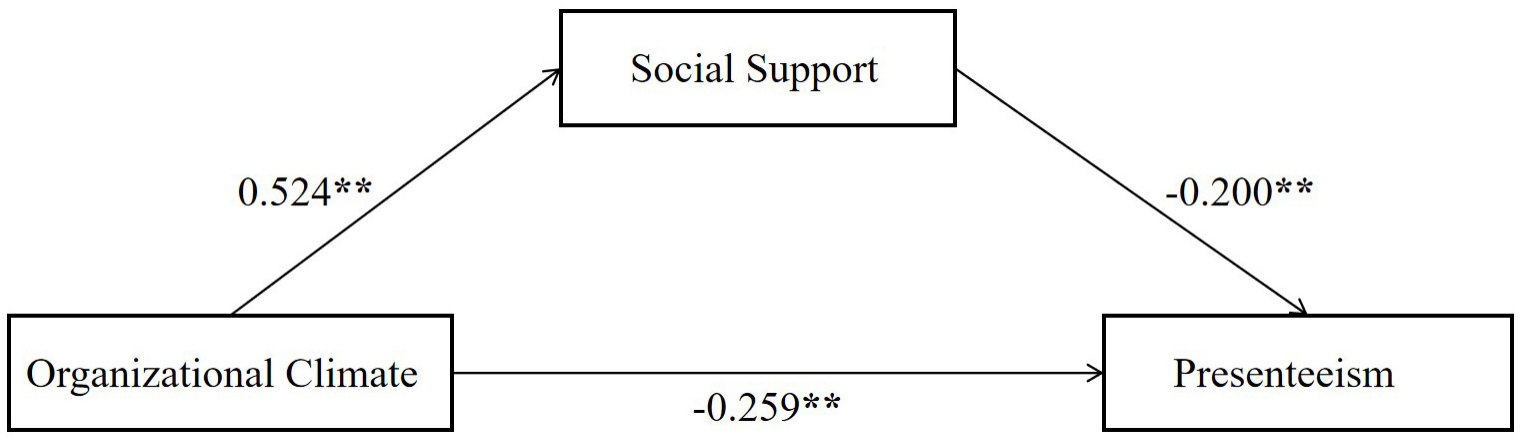
Statistical model of the mediating role of social support between organizational climate and presenteeism. ** *p* < 0.01.

After adjusting for physical health status and the provision of protective equipment, the model demonstrated a significant positive direct association between organizational climate and social support (β = 0.524, 95% CI: 0.422, 0.614, *p* = 0.001). Additionally, organizational climate maintained a significant negative direct association with presenteeism (β = −0.259, 95% CI: −0.413, −0.071, *p* = 0.006), and social support similarly exhibited a significant inverse direct association with the outcome variable (β = −0.200, 95% CI: −0.358, −0.048, *p* = 0.005). Bias-corrected bootstrapping analysis revealed a standardized indirect effect of −0.105 (95% CI: −0.197, −0.026, *p* = 0.005) linking organizational climate to presenteeism via social support, alongside a standardized total effect of −0.364 (95% CI: −0.494, −0.207, *p* = 0.003). Given that the 95% confidence intervals for both the direct and indirect pathways did not encompass zero, the findings indicate that social support partially mediates the association between organizational climate and presenteeism. This mediating pathway accounted for 28.85% of the total effect (Figure 2, Table 7).

**Table 7:** Bootstrap analysis of the mediating effect of social support between organizational climate and presenteeism.

Effect	Paths	Standardized effect (β)	*p*	95% CI
Direct	Organizational climate → Social support	0.524	0.001	[0.422, 0.614]
	Organizational climate → Presenteeism	−0.259	0.006	[−0.413, −0.071]
	Social support → Presenteeism	−0.200	0.005	[−0.358, −0.048]
Indirect	Organizational climate → Social support → Presenteeism	−0.105	0.005	[−0.197, −0.026]
Total	Organizational climate → Presenteeism	−0.364	0.003	[−0.494, −0.207]

CI, confidence interval.

## 4 Discussion

Grounded in the Job Demands Resources model, this cross-sectional study examined the associations among organizational climate, social support, and presenteeism among endoscopy nurses. The results indicated that both organizational climate and social support were negatively associated with presenteeism. Furthermore, social support was identified as a potential partial mediator in the association between organizational climate and presenteeism.

In the present study, the presenteeism levels reported by endoscopy nurses appeared slightly higher compared to previous findings among general clinical nurses (5). This relative elevation may be related to the specific operational demands of endoscopy units, where nurses routinely endure heavy procedure volumes and continuous exposure to occupational hazards (29, 30). Concurrently, the perceived organizational climate tended to be slightly lower than that observed in previous studies among general ward nurses (31). This slight difference is likely associated with the physical separation of endoscopy units from main hospital wards, an isolation that may contribute to frontline staff feeling disconnected from central management and broader institutional support systems. Notably, participants evaluated the hospital management dimension most poorly, suggesting a perceived misalignment between clinical productivity expectations and available administrative support (32). Furthermore, while the overall social support was comparable to broader healthcare samples, support was distinctly lacking for specific vulnerable subgroups, particularly single and childless nurses, as well as those in secondary level and below hospitals, which aligns with previous literature (33, 34). This deficiency highlights an unequal distribution of institutional resources, which may contribute to certain staff potentially more vulnerable to presenteeism.

The results revealed a negative association between organizational climate and presenteeism among endoscopy nurses, suggesting that a positive organizational climate is associated with a lower likelihood of presenteeism. Previous studies have consistently supported this association (35).According to the Job Demands Resources model, organizational climate functions as a crucial structural resource that may serve as a buffer against the impact of high clinical demands and occupational stress (36).In the intensive context of endoscopy units, a supportive environment may contribute to a lower perceived obligation to work while ill. Conversely, under heavy operational demands, nurses might perceive taking legitimate medical leave as disruptive to the workflow, which may result in a health requirement being perceived as a source of operational strain (7, 37).

The results revealed a negative association between social support and presenteeism, a finding consistently supported by previous literature (38). Extending prior research, the current study identified that social support may serve as a potential partial mediator in the association between organizational climate and presenteeism. This suggests that a positive organizational climate is associated with a lower likelihood of presenteeism, potentially by fostering robust peer support networks. Within the motivational process of the Job Demands Resources model, organizational climate functions as a foundational structural resource that may facilitate the accumulation of interpersonal resources like social support (36). Social support may serve as a critical psychological and operational buffer against occupational stressors (39). In high volume endoscopy settings, strong peer networks may facilitate the negotiation of shift replacements and enable nurses to prioritize recovery. The mediating pathway suggested by this study demonstrates how structural workplace resources are linked to protective interpersonal networks to support the management of presenteeism.

To effectively translate these findings into practice, nursing administrators could consider feasible, stratified interventions focused strictly on optimizing organizational resources and support structures. At the endoscopy unit management level, head nurses might enhance the work collaboration climate by optimizing existing workload distribution through regular team communications and trialing intra-shift dynamic rotations (40, 41). For instance, alternating frontline staff between high-intensity procedures such as ERCP assistance and lower-strain duties may provides practical physical relief (42). Concurrently, unit leaders could facilitate an informal peer-mentoring approach, pairing experienced endoscopy nurses with junior staff to provide continuous emotional support. Furthermore, managers might actively encourage staff to seek help when unwell, which could facilitate timely recovery and mitigate potential risks to patient safety associated with illness-related fatigue (43). At the institutional hospital policy level, administrators could bolster objective support by streamlining the administrative approval process for short-term sick leave and establishing fast-track medical consultation channels. Additionally, providing appropriate compensation or administrative flexibility for nurses who internally cover unexpected absences may serve as a tangible management resource (44). Ultimately, these grounded modifications focus directly on improving the organizational climate and social support, which may provide a realistic structural buffer against presenteeism to safeguard the quality of patient care.

## 5 Conclusions

This study indicates that organizational climate and social support are significant protective factors inversely associated with presenteeism among endoscopy nurses. The findings suggest that social support acts as a partial mediator in the association between organizational climate and presenteeism. These results highlight that addressing the issue of presenteeism requires targeted administrative strategies, rather than relying solely on the individual resilience of the nursing staff. By implementing practical structural modifications, nursing managers might transform informal peer help into reliable institutional resources. Specific stratified interventions, including dynamic intra-shift rotations, structured peer mentoring frameworks, targeted occupational health programs, and specialized reserve talent pools, may contribute to the better management of professional pressure. Ultimately, cultivating a supportive environment at both the unit management and institutional policy levels may be associated with a lower prevalence of presenteeism, which could subsequently support the sustainability of the nursing workforce and the safeguarding of patient safety in demanding endoscopic settings.

This study suggests that organizational climate and social support are inversely associated with presenteeism among endoscopy nurses. Furthermore, the findings indicate that social support may serve as a partial mediator in the association between organizational climate and presenteeism. These results highlight that addressing presenteeism may benefit from targeted administrative strategies rather than relying solely on individual resilience. Specific interventions stratified by unit and institutional levels could offer realistic structural buffers. Unit managers might explore optimizing workload distribution through dynamic shift rotations and facilitating informal peer mentoring to help provide continuous emotional support. Concurrently, hospital administrators could consider streamlining sick leave approvals, establishing expedited medical consultation channels, and providing appropriate compensation for nurses who cover unexpected absences internally. Ultimately, these grounded modifications aimed at improving the organizational climate and social support may contribute to the better management of presenteeism, thereby supporting the sustainability of the nursing workforce and the safeguarding of patient care quality.

## 6 Study limitations

Several limitations of this study must be acknowledged. First, utilizing a convenience sample restricted to hospitals within a single province introduces potential selection bias. This non-probability sampling method limits the external validity of the study, as the recruited cohort may not adequately capture the full demographic and occupational diversity of the broader endoscopy nursing workforce. Furthermore, regional variations in medical resource allocation, work environments, and career development opportunities across different provinces may contribute to differences in the levels of social support, organizational climate, and presenteeism among nurses. Consequently, the findings and observed associations may lack generalizability to endoscopy nurses operating in other regions with different socioeconomic contexts. Future multicenter studies should employ random sampling techniques across wider geographical areas to enhance the representativeness and external validity of the results.

Second, to resolve severe multicollinearity, variables including marital status, number of children, and hospital grade were excluded from the final regression models. Consequently, a limitation of this study is that the specific, independent associations between family structure, institutional tier, and nurses' presenteeism could not be evaluated. The final model primarily reflects the correlations involving health status, resource provision, and organizational psychosocial factors.

Third, the cross-sectional survey design limits data collection to a single time point, which precludes the determination of causal relationships among the variables. Longitudinal or interventional designs are warranted in future research to track dynamic changes and further elucidate the mediating mechanisms.

Finally, although social support was examined as a mediator, unmeasured confounding variables, such as individual personality traits, specific health conditions, and objective work stressors, were not controlled for in the current model. Subsequent research should incorporate objective measurement tools and account for a broader array of covariates to provide a comprehensive understanding of the factors associated with presenteeism in endoscopy nursing.

## Data Availability

The raw data supporting the conclusions of this article will be made available by the authors, without undue reservation.
